# Robotic Surgery in Gastrointestinal Surgery

**DOI:** 10.34133/2020/9724807

**Published:** 2020-12-04

**Authors:** Kenoki Ohuchida

**Affiliations:** Department of Oncology and Surgery, Kyushu University, Fukuoka, Japan

## Abstract

Robotic surgery is expanding in the minimally invasive treatment of gastrointestinal cancer. In the field of gastrointestinal cancer, robotic surgery is performed using a robot-assisted surgery system. In this system, the robot does not operate automatically but is controlled by the surgeon. The surgery assistant robot currently used in clinical practice worldwide is the leader-follower type, including the da Vinci® Surgical System (Intuitive Surgical). This review describes the current state of robotic surgery in the treatment of gastrointestinal cancer and discusses the future development of robotic systems in gastrointestinal surgery.

## 1. Introduction

In the past 20 years, there have been two big breakthroughs in the treatment of gastrointestinal cancer. One breakthrough is the evidence-based standardization of anticancer drug treatment, which was based on the results of many studies (including randomized controlled trials (RCTs)) performed throughout the world. The other breakthrough is the spread of minimally invasive surgery, including laparoscopic and thoracoscopic surgery [1]. Minimally invasive surgery is now widely used for gastrointestinal cancer, including esophageal cancer [2], gastric cancer [3, 4], hepatopancreatobiliary cancer [5, 6], and colorectal cancer [7, 8], and comprises endoscopic surgery such as laparoscopic surgery and thoracoscopic surgery. However, this spread of minimally invasive surgery differs from the abovementioned standardization of anticancer drug treatment because it is not based on firm evidence. The surgical methods and skills used during gastrointestinal cancer surgery differ between facilities and surgeons. Therefore, it is very difficult to compare the results such as the curability and complications of surgery, unlike the results of anticancer drug treatments. Although several reports, including RCTs, have been published in recent years [9, 10], only a few reports have provided definitive conclusions based on sufficient evidence. Despite this lack of definitive evidence for the advantages of minimally invasive surgery, the spread of this technique continues worldwide. This is possibly because the surgeons who perform minimally invasive surgery actually perceive the usefulness of such surgery.

**Figure 1:**
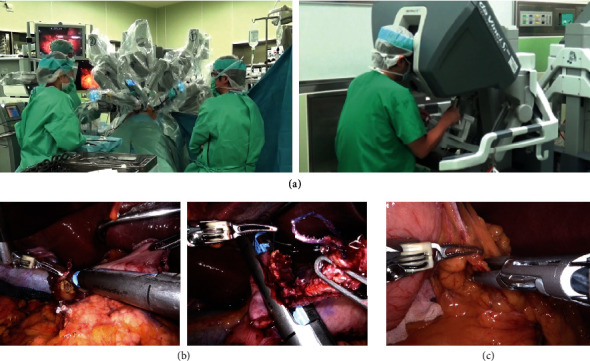
(a) The da Vinci Xi system; (b) the developed stapler for the da Vinci system; (c) the developed energy devices for the da Vinci system.

Today, the application of robotic surgery in gastrointestinal surgery is increasing as one of the most promising types of minimally invasive surgeries. In the field of gastrointestinal cancer, robotic surgery is performed using a robot-assisted surgery system. In this system, the robot does not operate automatically but is controlled by the surgeon. Currently, the surgery assistant robot used in clinical practice around the world is the leader-follower type of surgery assistant robot, the da Vinci® Surgical System (Intuitive Surgical, Sunnyvale, CA) (Figure 1(a)). The da Vinci system is approved for use in many countries. In Japan, the manufacture and sale of the da Vinci system as medical equipment were approved in November 2009, and robotic surgery using this system is now performed widely under public health insurance to treat esophageal cancer [11], gastric cancer [12], pancreatic cancer [13], and rectal cancer [14].

### 1.1. Current State of Robotic Surgery for Gastrointestinal Cancer

In 1997, the da Vinci Surgical System was introduced in the clinical setting for the first time in the world. The use of the da Vinci system for gastrointestinal surgery then spread in Europe and America, and the United States Food and Drug Administration approved this system in 2000. Now, more than 5,500 da Vinci systems have been introduced in more than 67 countries (source: Intuitive Surgical Investor Presentation).

In Japan, robotic surgery using the da Vinci system was carried out at Keio University in March 2000, and a clinical trial of a robotic surgery including cholecystectomy and Nissen fundoplication for gastrointestinal disease was performed in Kyushu University and Keio University until 2002 [15, 16]. In November 2009, the da Vinci system was approved for use in gastrointestinal surgery in Japan. The application of this robotic system is rapidly expanding in Japan.

Robotic surgery is currently performed worldwide to treat many types of gastrointestinal cancer, such as esophageal cancer [17], gastric cancer [18], and rectal cancer [19]. More recently, robotic surgery has been used to treat hepatopancreatobiliary cancer [20]. In 2014, a Japanese RCT performed to investigate the safety and utility of robotic surgery for gastric cancer reported that robotic surgery significantly decreased the postoperative formation of pancreatic juice fistula, which is one of the main complications after gastrectomy for gastric cancer [21].

In the early stage of introduction of robotic surgery in gastrointestinal surgery, simple operations such as cholecystectomy or Nissen fundoplication for hiatus hernia were performed using the robotic system to confirm its safety in the gastrointestinal field [15]. Robotic surgery now plays an important role in more complex surgery and has been widely applied in gastrointestinal surgery for various cancers [11, 13, 22].

### 1.2. Advantages of Current Robotic Surgery for Gastrointestinal Cancer

Surgery for gastrointestinal cancer requires the fine dissection of lymph nodes around the target organs. The present endoscopic surgery not only is a minimally invasive approach but also enables the fine and accurate dissection of lymph nodes under high-resolution imaging [23, 24], which may increase the curability. The use of endoscopic camera systems that provide full high-definition images is already spreading throughout the world. Furthermore, a novel camera system provides 4K and three-dimensional images [25, 26]. Therefore, endoscopy provides surgeons with a magnified view of the target tissues in the surgical field. The microscopic view of such camera systems enables surgeons to perform the fine and precise dissection of lymph nodes, which was difficult to perform under nonmagnified vision in laparotomy. However, this enhanced visual information requires more precise skill with endoscopic forceps. This precise manipulation can be achieved using the current robotic system [27]. The robotic system has articulated forceps, a filtering function to remove physiological vibration caused by the surgeon's hands, and a motion scaling function to reduce the movement of the surgeon's hands. Based on the magnified view provided by the camera system, these functions contribute to the achievement of more precise and accurate procedures that the surgeons could not have performed with their hands in laparotomy or with the usual forceps in endoscopic surgery. Fragile minute tissues tear and bleed when they are held by a human hand. In contrast, robot arms can easily hold such tissues without damage by maintaining the appropriate tension in the appropriate direction. The present robotic system equipped with these functions and three-dimensional full-high definition images enables surgeons to perform minute surgical procedures and contributes to the development of microsurgery, which was not possible using prior surgical techniques.

Several devices have recently been developed for use in robotic gastrointestinal surgery. In 2014, a software-regulated stapler was developed for the da Vinci system. Subsequently, the first robotic stapler for the da Vinci system, the EndoWrist Stapler, was introduced in Japan in 2016. The newest robotic stapler for the da Vinci system is the SureForm, which was introduced in Japan in 2019 (Figure 1(b)). This new SureForm stapling device has a system to monitor the tissue compression before and during firing and make automatic adjustments to form reliable staple lines. Furthermore, the SureForm stapler has a 120° cone of articulation, which makes it easy to maneuver the stapler within the limited space of the abdominal or thoracic cavity. Energy devices for robotic surgery have also been developed. The Vessel Sealer Extend (Figure 1(c): right panel), which has articulation and can seal and cut vessels with a minimum diameter of 7 mm, was previously frequently used for robotic gastrointestinal surgery. However, the tip size of the Vessel Sealer Extend was too large for fine dissection of lymph nodes in robotic surgery for gastrointestinal cancer. The recently introduced SynchroSeal (Figure 1(c): left panel; source: Intuitive Surgical Investor Presentation) has a fine curved jaw and can seal and cut vessels as small as 5 mm in diameter. Gastrointestinal surgeons in Japan may soon use such fine energy devices to perform more precise lymph node dissection during robotic surgery.

The current robotic system has fluorescence imaging. Using this function and indocyanine green injection, gastrointestinal surgeons confirm the blood supply of the organs around the anastomosis and confirm the lymphatic flow and lymph nodes around the tumors. Furthermore, the tumors themselves are identified using this fluorescence imaging when the tumors accumulate indocyanine green. Such real-time identification of important landmarks during robotic surgery contributes to improvements in safety and oncological curability.

### 1.3. Disadvantages of Current Robotic Surgery for Gastrointestinal Cancer

There are currently several problems with robotic surgery for gastrointestinal cancer. One of these problems is that it is difficult to provide firm evidence to show the usefulness of robotic surgery. A surgeon performing robotic surgery can intuitively feel its usefulness. However, to provide sufficient evidence of the effectiveness of robotic surgery, it is necessary to objectively show the advantages achieved in the treatment result, as has been done in research providing evidence for the effectiveness of anticancer drugs. It is easy to show the advantage of robotic surgery in terms of its minimal invasiveness compared with open surgery, but there is no difference in the invasiveness of robotic surgery in comparison with conventional endoscopic surgery [28]. Conversely, disadvantages such as prolonged operation time and increased medical costs can be easily shown as objective data [29]. In gastrointestinal cancer surgery, the precise lymph node dissection achieved during robotic surgery is expected to improve curability and reduce intra- and postoperative complications. Several studies have reported that robotic surgery reduces complications [10, 30, 31]. However, conventional endoscopic surgery seems to achieve sufficient lymph node dissection and radical curability without critical complications. Therefore, it may be difficult to demonstrate that robotic surgery is superior to endoscopic surgery.

One of the biggest problems with robotic surgery is that the surgeon operating the robot has no tactile sensation. Even in endoscopic surgery, some tactile sensation can be obtained through the forceps, although the degree of sensation is lesser than in open surgery. However, surgeons using the robotic system completely lose tactile sensation, which may lead to injury of soft organs, such as the small intestine, colon, and pancreas. At present, surgeons performing robotic surgery use visual information to compensate for this loss of tactile sensation. Especially in surgery for gastrointestinal cancer, it is necessary to complete the operation without damaging soft tissues and organs. Therefore, surgeons need to acquire the ability to use this visual information to compensate for tactile loss.

Another problem with robotic surgery is the potential for the arms to cause damage. The robotic third arm can hold soft organs for a long time without positional displacement. This function is very helpful to maintain the surgical field, but it is possible that localized ischemic changes may be induced by such a long duration of holding without tactile input. Furthermore, in robotic surgery for esophageal cancer, the articulated arms sometimes damage the chest wall that is out of the visual field. In this circumstance, visual information cannot compensate for the loss of tactile sensation. However, robots that can reproduce such tactile sensations are rapidly being developed in Japan and overseas [32, 33], and it is expected that they will be incorporated into clinical systems in the near future.

### 1.4. Future Prospects of Robotic Surgery for Gastrointestinal Cancer

Conventional robotic systems have a limited effective range of motion, and it is difficult to perform a wide range of operations. Using conventional robotic systems, it took a long time to set up and reposition the body and also to redock. Furthermore, there is interference between the arms inside and outside the body, and interference between the patient and the scope. Even when an assistant's port is inserted as a hybrid operation, the operation of the assistant's forceps is extremely limited due to the presence of the robotic arms. However, many of these issues have been improved by the recently introduced da Vinci Xi (Figure 1(a)). In the near future, it is expected that the size of the robotic arm and body will be further reduced and that the operation time and setting time will be further shortened.

It is desirable to remove a sufficient but minimal amount of the organ in cases of liver resection for liver cancer or pancreas resection for pancreatic cancer. The accurate removal of such a limited area requires extremely sophisticated intraoperative navigation technology, and it is necessary to obtain accurate positioning information about the forceps and scope. In conventional endoscopic surgery, it is usually difficult to obtain such information in the abdominal area. In contrast, robotic systems can easily and accurately provide information regarding the camera and arm positions, and the fusion of robot surgery and navigation surgery in this area is currently accelerating [34–36]. It is expected that a navigation system will be actively incorporated into the robotic system, leading to an innovative breakthrough.

**Figure 2:**
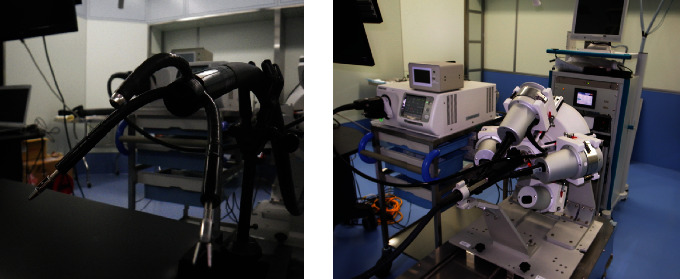
Robotic system for NOTES.

The Natural Orifice Transluminal Endoscopic Surgery (NOTES) robot is currently being developed in pursuit of less invasiveness than endoscopic surgery [37]. Although NOTES is an abdominal procedure, it does not require an incision in the skin or abdominal wall, and endoscopic surgical instruments and camera systems can be inserted through the mouth and the stomach wall, or through the vaginal or rectal wall. NOTES has been attracting attention as the ultimate minimally invasive surgery. However, because NOTES is performed through an orally accessible endoscope, all procedures are extremely difficult. Therefore, the application of NOTES in the treatment of gastrointestinal cancer is very complicated, and it is difficult to establish it as a standard treatment using ordinary instruments. To overcome this issue, a surgical assistance robot system for NOTES (a new suturing/closing device) is currently being developed [38]. Hashizume et al. at Kyushu University have also developed a robotic NOTES and have already successfully developed an endoscope equipped with robotic forceps (Figure 2). In addition, a surgeon from Kyushu University in Fukuoka has successfully completed an appendectomy using the robotic forceps of the robot NOTES in Thailand [39].

**Figure 3:**
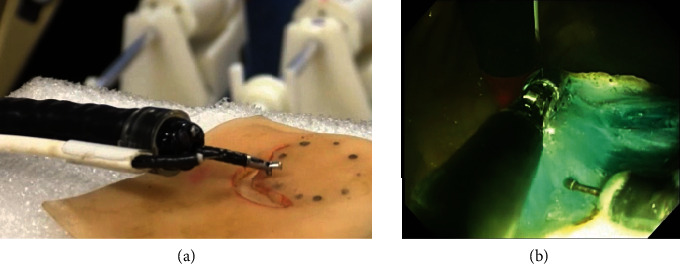
(a) Robotic system for NOTES; (b) ESD using the robotic system.

The advancement of the NOTES robot also requires the development of an endoscopic submucosal dissection (ESD) robot. ESD uses an upper gastrointestinal endoscope or a lower gastrointestinal endoscope without damaging the surface of the body. In this technique, the submucosal layer of the gastrointestinal tract is peeled off, and the entire layer is not resected. There is no postoperative pain, and organ function is preserved. Therefore, ESD is the least invasive treatment for early cancer, but it is also a very difficult procedure. In Japan, ESD is already widespread, but complicated procedures must be performed using a conventional scope with a limited angle operation of the tip of the scope and a treatment tool through one forceps hole. The degree of freedom of the operation is low and can take 10 hours or more to complete in some cases. Therefore, Kyushu University is developing a system with two arms that can bend from the distal end of a flexible endoscope to improve the operability of endoscopic treatment. To achieve this, a flexible endoscope with these two arms needs to be put together and passed through the patient's throat. Therefore, the cross-sectional diameter of this scope must be as small as possible. Hashizume et al. developed small flexible bending forceps for NOTES and ESD (Figures 3(a) and 3(b)) and have already verified its usefulness in animal experiments together with the intuitively operated controller developed at Kyushu University [40, 41]. Olympus Medical Systems also developed a platform equipped with two arms at the end of the endoscope under the project name ENDOSAMURAI [42]. Furthermore, the French Research Institute Against Digestive Cancer developed similar bending forceps under the project name Anubis in a joint study with Karl Storz. In addition, multiple flexible mirror robots such as the EndoMASTER (master and slave transluminal endoscopic robot) have been developed, mainly by the University of Singapore [43]. Thus, the development of devices in the field of endoscopic treatment using a flexible endoscope has gained attention around the world, and more robotic systems are expected to be developed in the future.

Single-port surgery (SPS) is also widely performed because it minimizes the damage to the abdominal wall [44–46]. In SPS, multiple forceps and a laparoscope are inserted through one hole, such as an umbilical port. This means that the operability of the forceps and scope is extremely poor, although the procedure is minimally invasive. In Japan, SPS for gastrointestinal cancer has been successfully performed without compromising curability and safety [47, 48], but it is not a common operation. One of the reasons that SPS is not used more frequently is that curability and safety are the top priorities in cancer treatment, while cosmetic issues are not so important. However, some types of gastrointestinal cancers are more likely to develop in young women, and it may be worthwhile to offer SPS to such patients. Robotic surgery is extremely useful for such SPS, and the development of surgical robots for SPS in this area is rapidly progressing [49]. The single-port da Vinci system has already been commercialized and used in gastrointestinal surgery [50], but the current system is not yet fully compatible with SPS for various gastrointestinal cancers. Further development will produce a robotic system for SPS in the future.

As mentioned above, one of the greatest advantages of robotic surgery is the extremely precise nature of the operation. Robotic systems that specialize in microsurgery are also being developed. The current da Vinci system is useful for microsurgery, but this system is generally designed for minimally invasive surgical environments such as endoscopic surgery. Robotic systems specialized for microsurgery will soon be developed and applied in a wide variety of situations, including laparotomy.

### 1.5. Limitations of the Present Review

Robotic systems in gastrointestinal surgery are rapidly progressing, and numerous studies are continually being published. Furthermore, many companies and researchers who develop robotic systems for clinical use do not provide sufficient information due to confidentiality obligations. Finally, the content of this review tended to include more information from Japanese studies because this information was easily accessible. Therefore, the content of the present review may involve bias and limitations.

## 2. Conclusions

Robotic surgery for gastrointestinal cancer is still in the introductory phase. In Japan, public health insurance covers the cost of robotic surgery for most gastrointestinal cancers, as well as for laparotomy and conventional laparoscopic surgery. Therefore, the choice of surgical treatment for each patient with gastrointestinal cancer includes laparotomy, conventional endoscopic surgery, and robotic surgery, and the selected type of surgery may depend on the facilities or the surgeons. These surgical methods need to be segregated to make effective use of limited medical resources when considering public insurance. For example, conventional endoscopic surgery should be selected in cases where it can be performed safely without a decrease in curability based on the patient's body type, body mass index, specific anatomical features, and degree of cancer progress. However, robotic surgery should be selected to further ensure safety and curability in cases with a high degree of technical difficulty. Such personalized treatment may be a promising means of selecting the most appropriate treatment.
